# Holding back the tiger: Successful control program protects Australia from *Aedes albopictus* expansion

**DOI:** 10.1371/journal.pntd.0005286

**Published:** 2017-02-13

**Authors:** Mutizwa Odwell Muzari, Gregor Devine, Joseph Davis, Bruce Crunkhorn, Andrew van den Hurk, Peter Whelan, Richard Russell, James Walker, Peter Horne, Gerhard Ehlers, Scott Ritchie

**Affiliations:** 1 Medical Entomology, Tropical Public Health Services Cairns, Cairns and Hinterland Hospital & Health Services, Cairns, Queensland, Australia; 2 Mosquito Control Laboratory, QIMR Berghofer Medical Research Institute, Royal Brisbane Hospital, Herston, Queensland, Australia; 3 Public Health Virology, Forensic and Scientific Services, Department of Health, Queensland Government, Archerfield, Queensland, Australia; 4 Biting Insect Technical & Extension Services, Nightcliff, Northern Territory, Australia; 5 Sydney Medical School, University of Sydney, Sydney, New South Wales, Australia; 6 Northern Australia Quarantine Strategy, Department of Agriculture and Water Resources, Cairns, Queensland, Australia; 7 Health Surveillance, Tropical Public Health Services Cairns, Cairns and Hinterland Hospital & Health Services, Cairns, Queensland, Australia; 8 College of Public Health, Medical and Veterinary Sciences, Australian Institute of Tropical Health and Medicine, James Cook University, Cairns, Queensland, Australia; North Carolina State University, UNITED STATES

## Abstract

**Background:**

The Asian tiger mosquito, *Aedes albopictus*, is an important vector of dengue, chikungunya and Zika viruses and is a highly invasive and aggressive biter. Established populations of this species were first recognised in Australia in 2005 when they were discovered on islands in the Torres Strait, between mainland Australia and Papua New Guinea. A control program was implemented with the original goal of eliminating *Ae*. *albopictus* from the Torres Strait. We describe the evolution of management strategies that provide a template for *Ae*. *albopictus* control that can be adopted elsewhere.

**Methodology / Principal findings:**

The control strategy implemented between 2005 and 2008 targeted larval habitats using source reduction, insect-growth regulator and pyrethroid insecticide to control larvae and adults in the containers. However, the infrequency of insecticide reapplication, the continual accumulation and replacement of containers, and imminent re-introduction of mosquitoes through people’s movement from elsewhere compromised the program. Consequently, in 2009 the objective of the program changed from elimination to quarantine, with the goal of preventing *Ae albopictus* from infesting Thursday and Horn islands, which are the transport hubs connecting the Torres Strait to mainland Australia. However, larval control strategies did not prevent the species establishing on these islands in 2010. Thereafter, an additional strategy adopted by the quarantine program in early 2011 was harborage spraying, whereby the vegetated, well shaded resting sites of adult *Ae*. *albopictus* were treated with a residual pyrethroid insecticide. Inclusion of this additional measure led to a 97% decline in *Ae*. *albopictus* numbers within two years. In addition, the frequency of container treatment was increased to five weeks between treatments, compared to an average of 8 weeks that occurred in the earlier iterations of the program. By 2015 and 2016, *Ae*. *albopictus* populations on the two islands were undetectable in 70–90% of surveys conducted. Importantly, a comprehensive surveillance network in selected strategic areas has not identified established populations of this species on the Australian mainland.

**Conclusions / Significance:**

The program has successfully reduced *Ae*. *albopictus* populations on Thursday Island and Horn Island to levels where it is undetectable in up to 90% of surveys, and has largely removed the risk of mainland establishment via that route. The vector management strategies adopted in the later years of the program have been demonstrably successful and provide a practical management framework for dengue, chikungunya or Zika virus outbreaks vectored by *Ae*. *albopictus*. As of June 2016, *Ae*. *albopictus* had not established on the Australian mainland and this program has likely contributed significantly to this outcome.

## Author summary

*Aedes albopictus* is a disease vector and biting nuisance of major public health concern. Established populations of *Ae*. *albopictus* were first recognised in Australia in 2005 after they were discovered on islands in the Torres Strait. Consequently, a control program was established in the same year to eliminate *Ae*. *albopictus* populations in the Torres Strait in order to reduce the risk of disease, as well as to prevent the mosquito from spreading to the mainland of Australia. In 2009, the goal of the program changed from elimination to quarantine (*cordon sanitaire)* focusing mainly on the inner islands of Thursday Island and Horn Island, which are the major population, administrative and transport centres linking the Torres Strait region to the Australian mainland. The *cordon sanitaire* strategy involved an integrated approach composed of harborage spraying, source reduction, insecticide treatment of containers, lethal tire piles, mosquito population monitoring and public awareness campaigns. Strategic improvements in management techniques led to a 97% decline in *Ae*. *albopictus* numbers on the two islands between 2011 and 2012. By 2015, the program had successfully reduced *Ae*. *albopictus* populations on Thursday Island and Horn Island to levels where the species was frequently undetectable, and had largely removed the risk of mainland incursion via that route. In 2016 the improved management strategies were also adopted to successfully control a dengue outbreak in which *Ae*. *albopictus* was the implicated vector on two outer islands of the Torres Strait.

## Introduction

The Asian tiger mosquito, *Aedes albopictus*, is a major public health concern. It is a vector of dengue, chikungunya and Zika viruses, and a potential vector of a wide range of other arboviruses [1–6]. Furthermore, *Ae*. *albopictus* is also considered one of the most significant nuisance mosquito species due to its high relative abundance and its aggressive day-biting behavior in peridomestic locations including backyards, leisure parks and gardens [7]. The combination of biting nuisance and vector status of *Ae*. *albopictus* underscores the importance of control of this species [8, 9].

An unprecedented global expansion of *Ae*. *albopictus* has occurred in the last four decades [10–12]. Although originally largely confined to the forests of south-east Asia, this highly invasive mosquito has readily adapted to diverse environmental conditions in urban and rural areas of both tropical and temperate regions [6, 12]. The movement of *Ae*. *albopictus* internationally has been mostly facilitated by the trade in used tires and other goods that are infested with eggs or larvae [13, 14]. Once introduced, *Ae*. *albopictus* has spread rapidly to occupy large areas of almost every region or country in which it had become established, including Florida [15], New Jersey [9] and Texas [16] in the USA, Spain [17], Italy [18], Cameroon [19], Papua New Guinea [20, 21] and many others.

**Fig 1:**
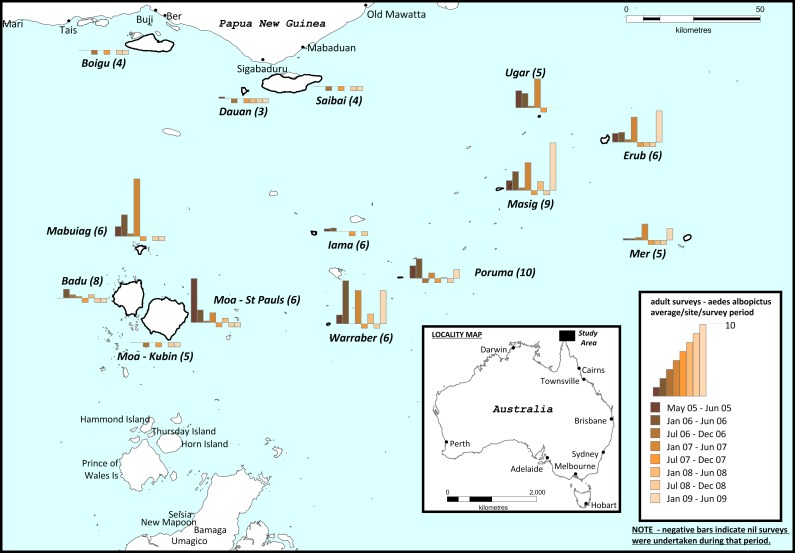
Mean human-bait sweep-net collections of adult *Ae*. *albopictus* from outer islands of the Torres Strait between 2005 and 2009. Numbers in parentheses indicate total number of adult mosquito surveys conducted on the island.

In Australia, *Ae*. *albopictus* was first detected on Masig (Yorke) Island in the Torres Strait in April 2005 [22]. The Torres Strait is a section of the eastern Arafura Sea approximately 150 km wide that separates the northernmost Australian mainland from the Western Province of Papua New Guinea (PNG) (Fig 1). It has at least 100 small islands, of which 17 are inhabited. A more detailed description of the islands is given by Ritchie et al. [22]. After the initial discovery of *Ae*. *albopictus*, a delimiting survey detected the species on nine other inhabited islands of the Torres Strait [22].

It was immediately recognised that the appearance of *Ae*. *albopictus* in the Torres Strait could lead to its subsequent establishment on the Australian mainland. A number of predictive models and expert reviews suggested that this mosquito could establish and survive in many populated regions of Australia, including those where *Aedes aegypti* does not occur [23, 24], and this would extend the risk of dengue, chikungunya and Zika transmission to these areas. Consequently, a control program funded by the Australian Government Department of Health was established in late 2005 to eliminate *Ae*. *albopictus* populations in the Torres Strait, and therefore reduce the risk of the mosquito being introduced to the mainland. The current paper describes the evolution of control strategies implemented during the 11 years that the program has been in operation. We discuss the challenges and limitations faced by the program, before outlining how it evolved into a very successful campaign, which has so far contained the infestation to the Torres Strait, protected that region’s major population centre, and mitigated the likelihood of *Ae*. *albopictus* colonizing the Australian mainland from that source.

## Methods

### Mosquito monitoring and control on the outer Islands

The control program, under the name *Aedes albopictus* Eradication Program (AAEP), was launched in late 2005 with the goal of eliminating *Ae*. *albopictus* from the Torres Strait [25]. A Technical Advisory Group of experts was established to regularly review efficacy of the program and make recommendations on its strategic direction. The program consisted of distinct surveillance and control components, and comprised a team of nine field staff based in Cairns, in north Queensland on the Australian mainland (Fig 1). The team travelled by air for two weeks of field work at a time, during which 2–4 islands were targeted. After the initial *Ae*. *albopictus* delimitation surveys across the Torres Strait in May 2005 [22], the islands were surveyed again in 2006 soon after the AAEP commenced, and at least once a year until September 2008. Surveys were also conducted on the islands of Masig, Poruma, Warraber, Erub and Mer in early 2009. Generally, frequency of control and surveillance visits was variable among islands, ranging between one and four per year including the dry season, depending on *Ae*. *albopictus* apparent densities and the size of the community.

On the islands, the team conducted source reduction, whereby any containers that could hold water and potentially support larval development were removed, destroyed, placed under cover, or treated with pellets or briquettes of the insect growth regulator s-methoprene. The s-methoprene was applied to smaller containers as pellets (40 g/kg a.i.; ProLink Pellets Mosquito Growth Regulator, Wellmark International, USA), at a rate of 1 pellet/L of estimated container volume. Larger containers, such as rainwater tanks and wells, were treated with ProLink XR Briquets (18 g/kg a.i.) applied at 1 briquet/5000 L water. Containers that could not be removed had their interior surfaces also sprayed with the residual pyrethroid, bifenthrin (Bistar 80SC, 80g/litre a.i., FMC Pty Ltd, Murarrie) to kill adults that come in contact with them [26]. Samples of larvae were collected from infested containers for species identification. Larvae were morphologically identified initially using the taxonomic keys of Rueda [27]. Due to overlapping morphology between larvae of *Ae*. *albopictus* and the endemic species, *Ae*. *scutellaris*, larvae suspected to be *Ae*. *albopictus* were submitted for identification using molecular methods [28, 29].

As part of the monitoring of mosquito populations on each island, the team also conducted human-bait sweep-net sampling for adult *Ae*. *albopictus* at potential harborage sites in the vicinity of residential properties and adjacent vegetation fringes. This is the quickest way to detect the presence of *Ae*. *albopictus*, and had been used since the delimiting surveys of 2005 [22]. Collectors with long-sleeved shirts and long trousers worked in pairs and spent five minutes at each site during daytime, collecting all mosquitoes flying around each person without allowing the mosquitoes to land or probe. Mosquito density was expressed as number of mosquitoes per collector per site [30].

An education and awareness campaign was conducted by the vector control field staff with support from the local Environmental Health Worker (EHW) stationed on each island. The EHW also facilitated yard access in the community. Residents were encouraged to routinely dispose of discarded water-holding containers, empty the non-essential water from domestic and peri-domestic temporary containers, or maintain mosquito-proof screens on permanent containers with essential water (e.g. rainwater tanks) around their homes. The campaign was supplemented with messages on local radio and in newspapers, as well as in schools.

### Focus on mosquito monitoring and control on Thursday Island and Horn Island

By mid-2008, *Ae*. *albopictus* populations still persisted on all infested islands because the elimination plan had become untenable due to logistical challenges associated with the need to repeat treatments on a more frequent basis and to the probable re-invasion of mosquitoes via the island network and traffic from PNG [28]. It was decided to change strategy, and focus predominantly on protecting the two inner islands of Thursday Island (population 3,100 with 650 properties) and Horn Island (population 700 with 170 properties). These are the major population, administrative and transport centres of the Torres Strait islands, and are the origin of almost all of the passenger and freight movements to the mainland. For that reason, they are considered to form the most likely regional origin for any mainland *Ae*. *albopictus* invasion.

Every year at least 460 vessels, mostly carrying cargo, sail from these islands to the mainland, especially to Cairns and Seisia. Establishment of *Aedes albopictus* on these destinations is highly likely once introduced, because both locations have environments which already support high populations of container-inhabiting mosquitoes such as *Ae*. *aegypti* and *Ae*. *notoscriptus*. In the Seisia and nearby communities, *Ae*. *scutellaris* is also widespread and it has generally similar ecological preferences to *Ae*. *albopictus*. Another pathway of incursion exists with at least 1,470 flights to Cairns from Horn Island annually. Both seaport and airport areas on Horn Island are bordered by bushland which is highly suitable as *Aedes albopictus* habitat, and potential larval habitats have repeatedly been identified in and around the port premises. Consequently, any significant populations of *Ae*. *albopictus* thriving in these areas would most likely lead to incursion and establishment on the Australian mainland.

At the time of adoption of the new strategy, known as a *cordon sanitaire*, the two islands were still free from *Ae*. *albopictus*. The insecticide for container treatment was changed to the pyrethroid lambda-cyhalothrin (Demand, 25g a.i./L, Syngenta Crop Protection, North Ryde) to provide greater residual activity, particularly for larval control [31, 32], although frequency of wet season retreatments was still variable, sometimes with more than eight weeks between treatments. Despite this re-focus of the AAEP, *Ae*. *albopictus* was discovered on Horn Island in March 2010 and on Thursday Island in December 2010, during yard inspections. The *cordon sanitaire* then changed from a strategy of exclusion to one of population suppression on these two islands, in order to minimise the pressure for a potential incursion to the mainland.

**Fig 2:**
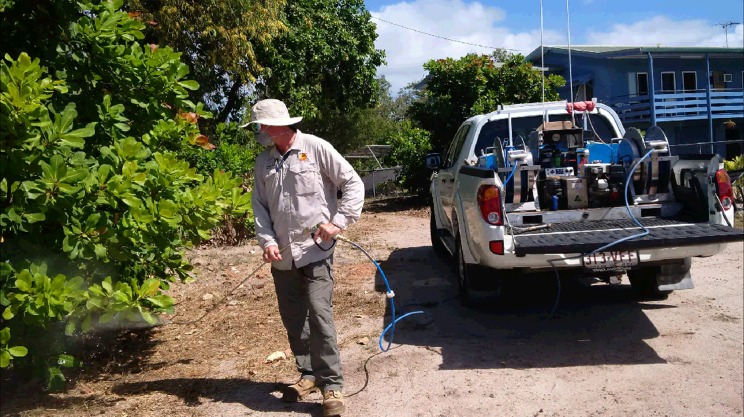
Harborage spraying using a vehicle-mounted unit to control *Ae*. *albopictus* on Thursday Island.

Starting from January 2011, the vector suppression efforts on Thursday Island and Horn Island, which had relied mainly on larval control, were supplemented with harborage spraying, targeting adults [30]. A limited field study on a backyard in China [33] had demonstrated that application of lambda-cyhalothrin to peri-domestic vegetation significantly reduced *Ae*. *albopictus* numbers for several weeks. Harborage spraying on Thursday Island and Horn Island involved the application of lambda-cyhalothrin to well-shaded vegetation below 2m height and leaf litter on the ground in locations identified as actual or potential resting sites for adult *Ae*. *albopictus*. Treatments were confined to backyard bushes and ~3-5m swath of fringing vegetation adjacent to residential and commercial properties. In general, the total treated area was less than 0.02% and 0.5% of the vegetated land area of Horn Island and Thursday Island respectively, minimising the impact on non-target fauna. This became a major additional component of the operations from that point forward. Between 2011–2012, harborage sprays were applied using a backpack mist-blower (Stihl SR420), but in 2013–2014 a tractor-mounted 200 L tank was used with a 50m long hose connected to a handheld lance fitted with a cone nozzle. From January 2015, the tractor-mounted tank was replaced by a more convenient high-pressure truck-mounted spray unit (Fig 2) (QuikSpray, QuikCorp Pty Ltd, Australia) with a handheld lance. The spray unit had been modified to replace its spray-gun with a longer lance and valve component from a pneumatic sprayer (B&G Equipment Company, Jackson, GA) for better direction of spray onto and under targeted foliage. In all cases, insecticide was diluted according to the label at 160 ml per 10 litres of water and applied almost to the point of run-off.

As a method to further protect port areas, lethal tire piles, consisting of 7–10 insecticide-treated car tires, were placed strategically ~20–50 m apart around seaport storage areas and airport buildings with the aim of attracting and killing gravid mosquitoes. Disused car tires are known to be attractive to container-inhabiting mosquito species [13, 32]. The tires were filled with water, treated with *s*-methoprene pellets, and sprayed internally with lambda-cyhalothrin (Demand 25g a.i./L). Treatments were repeated every 5–6 weeks in the wet season.

Property inspection, source reduction, container treatment, and larval surveillance were undertaken concurrently across all commercial and private residences on Thursday Island and Horn Island. For each inspection cycle, larval densities were expressed as number of positive containers per 100 houses (Breteau Index) for *Ae*. *albopictus* and *Ae*. *aegypti*. The interval between yard inspections (and reapplication of lambda-cyhalothrin to containers) was set at five weeks during wet seasons, with effect from January 2011. Completion of surveys sometimes overlapped between calendar months, and for graphing and analytical purposes, the data were allocated to the month that had more sampling days within a survey cycle. For adult surveillance, the teams conducted sweep-net sampling on 150 selected potential harborage sites on Thursday Island and 80 sites on Horn Island. This represented a doubling of the previous number of sites starting January 2011 to ensure comprehensive coverage within and around the community areas following the first detection of *Ae*. *albopictus* on Thursday Island in December 2010.

Completion of an operational cycle (control and surveillance across the two islands) during the wet season took 2–3 weeks. The following cycle would begin 2–3 weeks later. Consequently, each island was treated at five-week intervals. One dry season cycle was also conducted for 2 weeks between July and November, and primarily focused on yard inspections and treatment or disposal of potential, as well as perennial, larval habitats such as flower-pots, vases and rainwater tanks. Water-storage tanks and rainwater tanks were generally treated with s-methoprene briquettes if they were not effectively screened, but in 2015 and 2016 there was more focus on repairing or destroying the tanks where possible. Harbourage treatment was generally not conducted during the dry season because adult mosquitoes were undetectable at this time, and there are operational challenges due to the frequent strong winds typically experienced on the islands during that season, which may contribute to excessive spray drift.

### Mosquito monitoring on Hammond Island for comparison with Thursday Island and Horn Island

The effectiveness of the control operations was assessed by comparing mosquito populations on Thursday Island and Horn Island with those on Hammond Island, an inner island where *Ae*. *albopictus* was known to occur and for which control had never been conducted. Hammond Island is just 1 km from Thursday Island, and connected by a local ferry service and frequent local boat movements. It has a similar terrain and ecology to Thursday Island. Sweep-net surveys on Hammond Island were conducted on 10–15 selected sites with suitable potential habitat for *Ae*. *albopictus* on at least five occasions to coincide with wet season surveys on Thursday Island and Horn Island between 2012 and 2015.

### Mosquito monitoring on the mainland of Australia (Northern Peninsula Area, Townsville and Cairns)

To ensure that *Ae*. *albopictus* had not established on the north Queensland mainland, a long-term monitoring program was established in the Northern Peninsula Area (NPA) starting in January 2011. It consisted of 30 sticky ovitraps [34] strategically located throughout the communities of Seisia, New Mapoon, Bamaga, Umagico and Injinoo (Fig 1). The traps were checked and serviced weekly by a resident health technician in 2011 and 2012 before logistical issues with transport and time constraints forced discontinuation of the weekly trapping program. In addition, vector control officers from the Cairns team performed yard inspections and sweep-net surveys in the NPA communities over a two-week period once a year between February and May from 2011 to 2016. On every occasion, a total of 700 properties was inspected and sweep-net sampling was conducted on 110 selected sites across the five communities.

Other *Ae*. *albopictus* monitoring activities on the mainland of Australia occurred in the high-risk *Ae*. *aegypti*-infested tropical towns of Cairns and Townsville. These are the cities that receive much of the air and sea traffic that originates in the Torres Strait and parts of southeast Asia where *Ae*. *albopictus* is widespread. The monitoring involved up to180 mosquito traps in selected residential and industrial areas being checked weekly for more than seven years. The traps included sticky ovitraps assembled from locally-acquired materials or Gravid Aedes traps (GAT) [35] and Biogents Sentinel (BGS) traps (Biogents AG, Germany). Additional BGS traps and sentinel tire traps for larval sampling were deployed at the Cairns port areas where vessels from the Torres Strait moored. These have been monitored weekly by biosecurity personnel for at least the last 15 years. Furthermore, *ad hoc* surveys were conducted each year as part of dengue control interventions in Cairns, Townsville and surrounding areas.

## Results

### Mosquito monitoring and control on the outer Islands

The number of outer islands found to be infested with *Ae*. *albopictus* rose from 10 in 2005 to 13 in 2006, with detections on Badu, Boigu and Moa islands. Periodic sweep-net surveys conducted up to 2009 revealed spatial and temporal variability of *Ae*. *albopictus* populations among the islands, with the highest adult densities recorded on Masig, Warraber, Mabuiag, Erub and Ugar (Fig 1). Larvae of *Ae*. *albopictus* were detected through yard inspections on all 13 outer islands where adults had been detected. There were no detections of *Ae*. *albopictus* larvae or adults on Saibai Island and Hammond Island during this period. Overall, the control efforts were not able to provide sustained suppression of *Ae*. *albopictus* on the infested islands. Furthermore, genetic studies conducted on populations from the Torres Strait, Papua New Guinea and Indonesia suggested that if the species was eliminated, it would soon be re-introduced from an infested location [28].

### Mosquito monitoring and control on Thursday and Horn Islands

Before *Ae*. *albopictus* was detected on Horn Island and Thursday Island, survey results in 2009 and 2010 showed that the islands had pre-existing populations of other container-inhabiting species, including *Ae*. *aegypti*, *Ae*. *scutellaris* and *Culex quinquefaciatus*. Thursday Island had high densities of *Ae*. *aegypti* (BI ≥ 55 in March 2010) and had experienced dengue outbreaks due to this species previously [36, 37].

**Fig 3:**
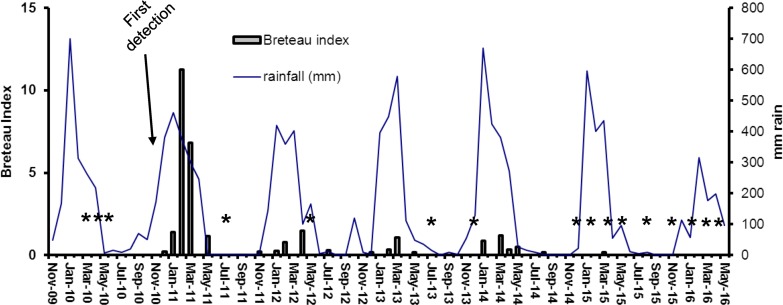
Prevalence of containers with *Aedes albopictus* larvae on Thursday Island, showing progressive decline over the period 2011–2016. Breteau Index represents number of positive containers per 100 houses inspected. Stars indicate surveys with nil detection.

**Fig 4:**
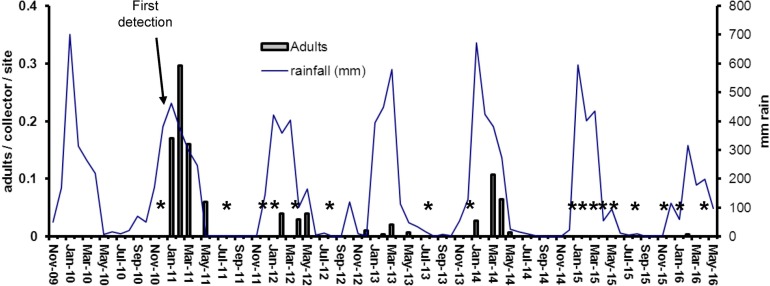
Mean sweep-net collections of *Ae*. *albopictus* adults on Thursday Island 2011–2016. Periodic five-minute collections were conducted on up to 150 selected sampling sites. Stars indicate surveys with nil detection.

**Fig 5:**
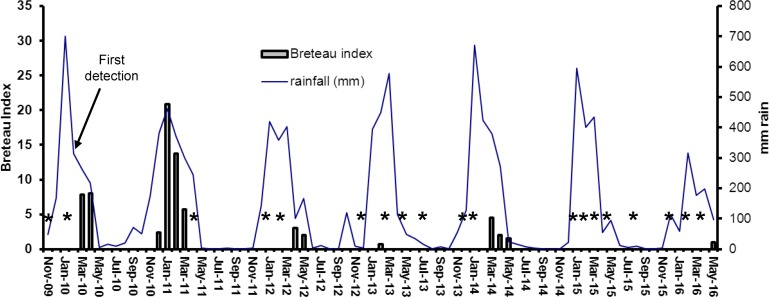
Prevalence of containers with *Ae*. *albopictus* larvae on Horn Island, showing population decline over the period 2011–2016. Breteau Index represents number of positive containers per 100 houses inspected. Stars indicate surveys with nil detection.

**Fig 6:**
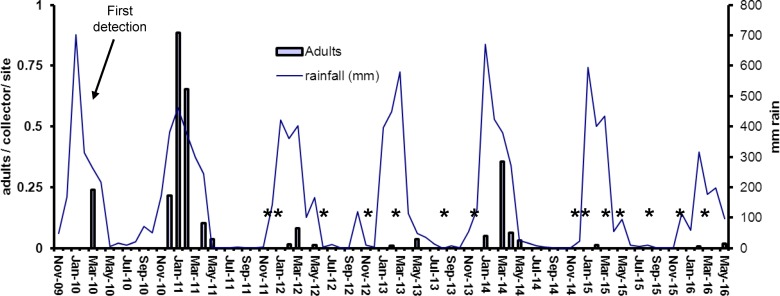
Mean sweep-net collections of *Ae*. *albopictus* adults on Horn Island over the period 2010–2016. Periodic five-minute collections were obtained from 80 sampling sites. Stars indicate surveys with nil detection.

During the first wet season after initial detections of *Ae*. *albopictus* infestations on TI and HI, the populations of this species increased very rapidly (Fig 3–Fig 6). Densities on both islands were highest in January-March 2011 (Fig 3 and Fig 5), with BI’s up to 21 and adult densities up to 0.9 per collector per sampling site (Fig 4 and Fig 6). When control efforts were intensified, starting in 2011 with the adoption of the harborage spraying strategy as an additional control tool, the BI’s declined by more than 80% in the 2012 wet season and by 90% in the 2013 wet season. With sustained suppression effort, only one positive container was found on TI and two on HI throughout the 2015 and 2016 wet seasons in which there had been 10 cycles of island-wide house to house yard inspections. Comparison of mean number of *Ae*. *albopictus*-positive containers detected on Thursday Island during peak wet-season conditions (January-April), showed statistically significant differences between year 2011 and each of the years 2012–2016 (Independent T-tests; p<0.05, df = 7).

The vector control team generally identified and treated at least 3,000 potential and actual larval habitats during each cycle of yard inspections across the two islands. Containers encountered included buckets, tires, wheel-barrows, watering cans, pot-plant bases, bird-baths, bowls, pots, portable cooler boxes, ice-cream containers, take-away food containers, plastic sheets, tarpaulins, buckets of ornamental plant cuttings, building materials, lawn-mower catchers, drums, drain sumps, fence posts, disused household appliances such as washing machines and fridges, boats and discarded car bodies. Natural larval habitats, such as coconut shells, bromeliads and palm fronds were also recorded. General observations indicated no consistent reduction in the number of containers recorded during yard inspections. In many cases, larger items, such as boats, car bodies and disused appliances were always found at the same places as before, and items that often reappeared after removal included palm fronds, buckets, discarded take-away food containers and items of household rubbish. In 2015, most of the car bodies and some of the disused appliances were removed from the community through the efforts of the Torres Shire Council.

**Fig 7:**
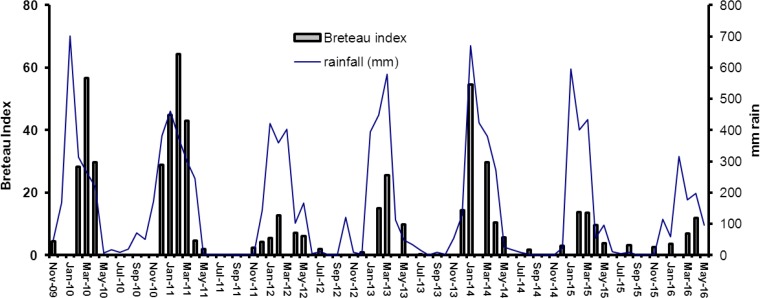
Prevalence of containers with *Ae*. *aegypti* larvae on Thursday Island for the period 2009–2016.

Interestingly, the impact of the control program on containers positive for *Ae*. *aegypti* (Fig 7) was not as appreciable as it was on *Ae*. *albopictus*, most likely because none of the control strategies specifically targeted the adult resting sites of *Ae*. *aegypti*, and the surviving adults potentially utilised cryptic larval habitats.

### Mosquito monitoring on Hammond Island for comparison with TI and HI

**Fig 8:**
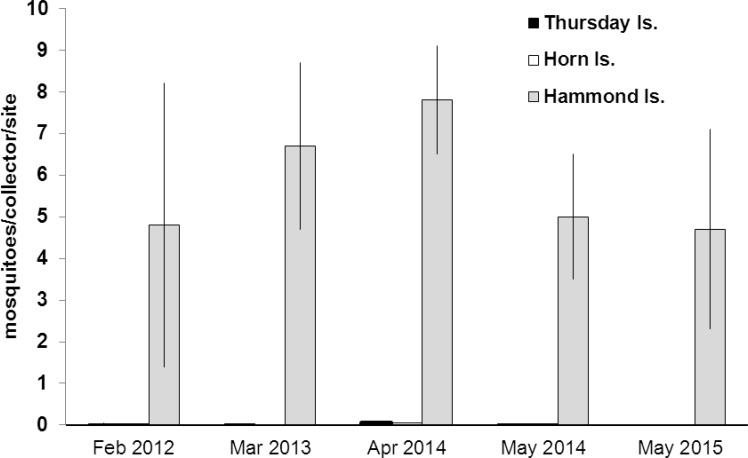
Mean (+SE) sweep-net collections of *Ae*. *albopictus* adults obtained from selected sampling sites on Horn, Thursday and Hammond islands at various times between 2012 and 2015. There were no vector control activities on Hammond Island. Previous survey on Hammond had been conducted in 2009 and did not detect any *Ae*. *albopictus*.

Occasional surveys over several years on nearby Hammond Island showed that *Ae*. *albopictus* was well-established by February 2012, and would have most likely invaded the island at some point between 2009 and 2011. Densities of *Ae*. *albopictus* averaged 5–8 adults per collector per site in five-minute sweep-net collections on each survey conducted during the wet season between 2012 and 2015 (Fig 8). In contrast, densities on TI and HI in the same months were less than 0.1 per collector per inspected site. This difference demonstrated the considerable impact of the suppression program on TI and HI. There was no detection of *Ae*. *aegypti* on Hammond Island.

### Mosquito monitoring on the mainland of Australia

The mainland surveys in the NPA between 2011 and 2016 detected a variety of species which have been recorded from the area previously, including *Ae*. *aegypti*, *Ae*. *notoscriptus*, *Ae*. *scutellaris*, *Ae*. *tremulus*, *Ae*. *vigilax*, *Cx*. *quinquefaciatus*, *Cx*. *annulirostris*, *Verrallina* spp. and *Tripteroides* spp. Previously, there had been a single detection of *Ae*. *albopictus* in the NPA, when larvae were found in a small container in the New Mapoon community in 2009 during yard inspections conducted in response to a local dengue importation [38]. The container was removed, together with other potential larval habitats during the dengue response. This was an isolated incident and there have been no further detections on the NPA.

## Discussion

One of the key outcomes of the AAEP is that, although *Ae albopictus* has been widely established in the Torres Strait for over 10 years, it does not appear to have spread from there and, as of June 2016, no established populations of *Ae*. *albopictus* have been discovered anywhere on the mainland of Australia. The Torres Strait to mainland route remains a major threat for *Ae*. *albopictus* importation, given that containers or equipment that have spent time outdoors exposed to rain, could be carrying desiccation-tolerant eggs [13, 39], and can then be transported to the mainland as returning construction or maintenance equipment or as personal effects. However, mosquito suppression at the main transit hubs of TI and HI appears to have been an effective strategy in reducing this risk.

The *cordon sanitaire* strategy is an integrated approach composed of harborage spraying, source reduction, insecticide treatment of containers, lethal tire piles, mosquito population monitoring and public awareness campaigns supported by local authorities and local media. The consistently low densities of *Ae*. *albopictus* on TI and HI recorded from March 2011 onwards have demonstrated that harborage spraying with lambda-cyhalothrin appears to be the most important component of the intervention program. Smaller-scale field studies in backyard scenarios elsewhere have also shown considerable reductions in *Ae*. *albopictus* numbers after application of residual pyrethroids to vegetation [33, 40, 41]. Other studies have also reported that this method is even more efficacious when coupled with intensive source reduction. For example, in the Caribbean, a control program on Grand Cayman Island managed to effectively suppress *Ae*. *albopictus* densities between 1997–1999, restricting the BI to below 0.65 in George Town, when control methods included both house-to-house source reduction and residual application of lambda-cyhalothrin to nearby vegetation and walls [42]. However, when strategies were changed due to financial constraints in 1999 to concentrate primarily on larval control, the BI rose more than ten times to 6.9 within the following two years, and the infested area more than doubled in the same period.

Considerable effort in the Torres Strait was made to empower the local communities to eliminate containers through public announcements, communication via the media and with direct communication with householders. However, the continued presence of many established and new potential mosquito-containers on properties showed that the public was possibly overwhelmed by the amount of effort required in keeping the yards free of water-holding containers, especially in the wet season when rain readily fills any exposed containers. In parts of New Jersey, public education was similarly found to be insufficient in motivating residents to significantly reduce backyard mosquito-larval habitats [43]. However, public education in the Torres Strait was still partly successful, because the majority of residents co-operated with the AAEP control teams and allowed them access to their properties.

While access restrictions (residents not home during working hours, vacant and locked properties or access denial from residents) can sometimes make it difficult to inspect and treat potential habitats [16, 44], this was not a major issue on Thursday Island and Horn Island because the AAEP personnel had excellent advocacy support from local government leaders. The program also performed under a well-publicised legislative arrangement in which control personnel were authorised to enter yards, even if residents were not home. However, the communities were aware of the benefits of such a program, so there were less than 1% refusals, and thus, legislative powers rarely had to be enforced.

Although the precipitous decline of *Ae*. *albopictus* on TI and HI can be attributed to the integrated control strategies of the AAEP, it is important to acknowledge that natural ecological factors contributed to that downward pressure, at least seasonally. The Torres Strait experiences low rainfall and humidity between June and November (Fig 3), and *Ae*. *albopictus* populations can fall to relatively very low levels. That respite during the dry season has formed a key part of the overall strategy and kept annual control costs down. However, in the absence of any control program, *Ae*. *albopictus* can recover quickly during the wet season and can reach very high densities (as evidenced by mosquito collections on Hammond Island (Fig 8) and the other outer islands). While the results demonstrate the success of the *Ae*. *albopictus* suppression program on TI and HI, they also indicate that the threat of reinvasion from other infested islands remains high.

There is still a risk that *Ae*. *albopictus* may be introduced via another mainland port from other international ports. For instance, within the last five years this species has been intercepted at international airports in Perth, Melbourne and Darwin, as well as seaports in Townsville, Darwin, Perth, Melbourne, Cairns and Brisbane. The interceptions involved adults caught in surveillance traps at the ports and sometimes larvae, pupae or adults detected on vessels or cargo arriving from places like Indonesia and PNG [45, 46]. This is not surprising, as there had been several similar incidents at these and other ports well before *Ae*. *albopictus* infested the Torres Strait islands [46–48]. In response to an interception at the port area, it is standard practice for emergency mosquito control and surveillance operations to be conducted immediately within 1 km of the port. All responses have been successful so far, with no evidence of establishment after treatment [22, 25]. An importation into Melbourne in 2012 was particularly concerning as it occurred via a shipment of *Dracaena* spp. (lucky bamboo) to a nursery [25]. Fortunately, the infestation was confined to a quarantine-approved premise and there was no evidence that it had breached this containment. As of June 2016 there was no evidence to suggest *Ae*. *albopictus* has become established on the Australian mainland.

Although the original objective of the AAEP was the elimination of *Ae*. *albopictus* from all of the Torres Strait islands, for a number of reasons, this became untenable given the limited funding available, which limited the human resources available and the amount that could be spent on logistics (airfares to and between the islands, accommodation for the project workers, etc), and resulted in a low frequency of surveillance and control visits to each island. Working in the outer islands presented further logistical problems, such as the lack of accommodation, and the costs and delays in transporting insecticides and equipment by sea. Furthermore, population genetics studies [28] indicated that there was a high potential for reinvasion due to sea traffic between islands, and also from PNG and the Indonesian archipelago. Thus, the implementation of a *cordon sanitaire* on the two inner islands offered the most effective utilisation of limited resources.

The limited impact in suppressing the populations on the outer islands in the first three years of the program appears to have been the result of using larval control as the primary control strategy, and insufficient frequency of insecticide reapplications. Although *Ae*. *albopictus* is strongly associated with peri-domestic environments [49], house-to-house source reduction and container applications of insecticides have limited impact as a sole intervention method, due to the ubiquitous and often cryptic larval habitats of this species [50] and, in the case of the Torres islands, the high rate of container replacement. In the Torres Strait, high-set water-storage tanks and rainwater tanks, as well as subterranean sites, are difficult to access and treat, whilst refuse, such as take-away containers, is continuously being produced and replaced, providing new untreated habitats for *Aedes* spp. In the successful *Ae*. *aegypti* elimination programs in the Northern Territory of Australia, it was concluded that at least a 6-week reapplication of residual insecticides to containers was necessary, in light of the effective length of insecticide activity and the continual production of new containers [26, 32]. Therefore, the adoption of lambda-cyhalothrin as the primary insecticide for application in containers was more effective, as the insecticide would last at least 6 weeks and possibly up to 9 weeks [32], compared to bifenthrin, which remains active for only approximately 2 weeks as a larvicide [31].

There were a number of methods employed for eliminating *Ae*. *aegypti* during the incursions into the Northern Territory, including application of *s*-methoprene, and residual pyrethroids to containers, which targeted both larvae and ovipositing adults, household bleach to kill eggs, and treatment of adult harborage areas adjacent to houses. It was concluded that the use of one method alone would not have led to success [26].

The suppression strategies of the AAEP did not have a pronounced effect on *Ae aegypti* populations on TI. This is probably because harborage spraying targets the vegetated resting sites of *Ae*. *albopictus*, and is not as relevant to the control of *Ae*. *aegypti* with its more endophilic, domestic resting behaviours (i.e. patio furniture, garden sheds and especially the interior of houses [51]. Furthermore, cryptic larval habitats utilised by *Ae*. *aegypti*, such as un-located subterranean disused septic tanks and wells, may have enabled the greater survival of this species during the harsh dry season, before proliferation during the wet season.

The evolution of the control programme from one of elimination to that of a *cordon sanitaire* approach, coupled with introduction of the harborage spraying component, has resulted in the successful suppression of *Ae*. *albopictus* populations on the primary transport hubs of the Torres Strait. This has reduced the risk of this species being introduced onto the Australian mainland via this route. This integrated strategy provides a template that can be followed for control of this species on the Australian mainland, should it be intercepted or become established. Indeed, recent interceptions of this species in Cairns, from origins other than the Torres Strait, have utilised strategies implemented during the AAEP. This strategy also provides a practical solution for effective management of dengue, chikungunya or Zika outbreaks in areas where *Ae*. *albopictus* is the primary vector. Indeed, the strategy was used in March 2016 to control a dengue outbreak on Erub and Badu Islands, leading to a rapid decline in cases and cessation of transmission [52]. The islands had dense populations of *Ae*. *albopictus* with no detection of *Ae*. *aegypti*, and this further demonstrated the potential public health risk due to *Ae*. *albopictus* if the species were to spread to southern parts of Australia where dengue vectors do not currently exist.
